# Editorial: Medicinal *Cannabis*: Evolution of therapeutic use, future approaches and other implications

**DOI:** 10.3389/fphar.2022.999068

**Published:** 2022-08-25

**Authors:** Paola Brusa, Francesca Baratta, Massimo Collino, Shimon Ben -Shabat

**Affiliations:** ^1^ Department of Drug Science and Technology, University of Turin, Torino, Italy; ^2^ Department of Neurosciences Rita Levi Montalcini, University of Turin, Turin, Piedmont, Italy; ^3^ Department of Clinical Biochemistry and Pharmacology, Ben-Gurion University of the Negev, Beersheba, Southern, Israel

**Keywords:** *Cannabis*, formulations, effectiveness, medical use, clinical trials

*Cannabis* has been used in even the oldest traditional medicines available. In the last century, negative attention has prevailed regarding the psychotropic and abuse potential. For this reason, *Cannabis* has been banned and declared illegal in many countries. In recent years, however, there has been a more in-depth evaluation of the legalization of cannabinoids for medical use in several countries following heightened media attention and reports of effectiveness, although not always thoroughly backed up by scientific evidence. The official introduction of pharmaceutical-grade *Cannabis* inflorescences for medicinal purposes has allowed physicians and pharmacists, to prescribe and prepare several *Cannabis* preparations legally. Such products are currently being administered to patients without their efficacy being evaluated in controlled studies: for each patient the composition and route of administration may differ. In addition, many advanced administration systems have been developed or are still under development, but few clinical trials have been completed.

In this context, this Research Topic focused on the in-depth analysis of the legal, technological and pharmacological aspects related to the medical use of *Cannabis*-based formulations.

Anil et al. have directed their research specifically on the activity of *Cannabis* for medical use in the context of inflammatory processes. Although activities in this area are plausible, the high number of active molecules produced by *Cannabis* and simultaneously administered through the extractive products normally used in therapy, has not yet made it possible to identify their specific mechanisms of action. Once the modalities of action of the active molecules have been clarified, it might be of interest to use purified mixtures to obtain a more significant activity potentially (Anil et al.).

Specific literature reviews were then done for some pathologies such as when Xin et al. investigated the potential therapeutic effect of CBD in bone diseases. Even in this case, further studies are needed to evaluate the benefits and risks of cannabinoids’ use (Xin et al.).

A large part of the clinical research relating to *Cannabis* for medical use concerns its use in the context of diseases of the central nervous system. Ortiz et al. examined evidence supporting the therapeutic utility of cannabinoids for treating neurodegenerative diseases, pain, mood disorders, and substance use disorders. Important considerations were also made on the methods of formulation and the routes of administration (Ortiz et al.). Lacroix et al. Also considered *Cannabis* in neurological disorders stressing that currently most of the scientific data supports the potential therapeutic use of *Cannabis* but, as much as patients request it, the knowledge is still too little in-depth. It is therefore certainly urgent to manage clinical trials to provide stronger and safer evidence (Lacroix et al.).

Procaccia et al. discussed how phytocannabinoid profiles differed between plants according to chemovar types and examined the main factors influencing the accumulation of secondary metabolites in the plant, including genotype, growing conditions, processing, storage and the delivery route; the authors highlighted how these factors make the use of *Cannabis* in therapy highly complex (Procaccia et al.).

In addition to the more well-known compounds such as THC and CBD, *Cannabis* produces over 120 other phytocannabinoids. The use of THC is associated with acute psychotropic effects that could potentially be avoided considering that minor cannabinoids and their chemical counterparts could offer the same potential benefits without the same adverse effects. In this regard, Walsh et al. reviewed the literature to provide an overview of the endocannabinoid system, phytocannabinoid biosynthesis and a discussion on molecular pharmacology. Potential therapeutic uses of minor cannabinoids underlining that future studies will have to rigorously evaluate these compounds’ risk/benefit *ratio* (Walsh et al.).

The interest in molecules other than cannabinoids such as terpenes is certainly relevant. This interest has grown even greater since the possibility of an “entourage” effect between the active molecules of *Cannabis* has been postulated. Accordingly, Finlay et al. in their study examined whether some terpenes acted directly on cannabinoid receptors. From the results obtained, it was not possible to exclude the existence of an entourage effect. Still, this cannot be linked to a direct action of the terpenes on the cannabinoid receptors. However, the pharmacological mechanism underlying this substances activity remains to be thoroughly investigated (Finlay et al.).

Maayah et al. pointed out that full-spectrum Cannabis extracts have been used in clinical trials to treat various diseases. However, despite their efficacy, their potential use in therapy may be limited by possible behavioural side effects. These researchers then successfully worked on experimental animals to identify a panel of blood metabolites predicting behavioural effects (Maayah et al.).

Pennypacker et al. have evaluated whether the products available on the market in the United States of America are consistent in the concentration of cannabinoids, with the literature indications for use in therapy. Overall, the results of this study have been defined by the authors as alarming as current product offerings do not reflect scientific evidence (Pennypacker et al.).

In the regulatory context MacPhail et al. have analysed the trend of prescriptions in Australia over the last 5 years, noting a substantial increase in prescriptions over time that does not actually reflect a worsening of the pathological conditions of the population but rather a greater prescription linked to greater knowledge and acceptance of this type of therapy (MacPhail et al.).

As regards the use in therapy of medical Cannabis, the current regulations have been analysed by Baratta et al. in those countries where clinical studies have recently been conducted. The results of the trials have been crossed with the pathologies for which the current legislation provides that it is possible to prescribe *Cannabis* allowing relevant considerations (Baratta et al.).

From all the publications collected, it is clear that there is a great interest in the enormous potential of *Cannabis* in the medical field but also a widespread awareness of the extreme need to conduct in-depth research that clarifies the mechanisms of action of the quantity of components present in the phytocomplex of this plant species.

